# Does Delayed Cord Clamping Improve Long-Term (≥4 Months) Neurodevelopment in Term Babies? A Systematic Review and a Meta-Analysis of Randomized Clinical Trials

**DOI:** 10.3389/fped.2021.651410

**Published:** 2021-04-12

**Authors:** Serena Xodo, Luigi Xodo, Giovanni Baccarini, Lorenza Driul, Ambrogio P. Londero

**Affiliations:** ^1^Clinic of Obstetrics and Gynecology, University Hospital of Udine, Udine, Italy; ^2^Department of Medical Area (DAME), University of Udine, Udine, Italy; ^3^Laboratory of Biochemistry, Department of Medicine, University of Udine, Udine, Italy; ^4^Ennergi Research, Lestizza, Italy

**Keywords:** delayed cord clamping, neurodevelopment, placental transfusion, third stage of labor, cord clamping time

## Abstract

**Background:** Recently, the literature suggested that placental transfusion facilitated by delayed cord clamping (DCC), besides having benefits on hematological parameters, might improve the infants' brain development.

**Objective:** The present review primarily evaluates the Ages and Stages Questionnaire (ASQ) total score mean difference (MD) at long-term follow-up (≥4 months) comparing DCC (>90 or >180 s) to early cord clamping (ECC). Secondary aims consisted of evaluating the ASQ domains' MD and the results obtained from other methods adopted to evaluate the infants' neurodevelopment.

**Methods:** MEDLINE, Scopus, Cochrane, and ClinicalTrials.gov databases were searched (up to 2nd November 2020) for systematic review and meta-analysis. All randomized controlled trials (RCTs) of term singleton gestations received DCC or ECC. Multiple pregnancies, pre-term delivery, non-randomized studies, and articles in languages other than English were excluded. The included studies were assessed for bias and quality. ASQ data were pooled stratified by time to follow up.

**Results:** This meta-analysis of 4 articles from 3 RCTs includes 765 infants with four-month follow-up and 672 with 12 months follow-up. Primary aim (ASQ total score) pooled analysis was possible only for 12 months follow-up, and no differences were found between DCC and ECC (MD 1.1; CI 95: −5.1; 7.3). DCC approach significantly improves infants' communication domains (MD 0.6; CI 95: 0.1; 1.1) and personal-social assessed (MD 1.0; CI 95: 0.3; 1.6) through ASQ at 12 months follow-up. Surprisingly, the four-month ASQ personal social domain (MD −1.6; CI 95: −2.8; −0.4) seems to be significantly lower in the DCC group than in the ECC group.

**Conclusions:** DCC, a simple, non-interventional, and cost-effective approach, might improve the long-term infants' neurological outcome. Single-blinding and limited studies number were the main limitations. Further research should be performed to confirm these observations, ideally with RCTs adopting standard methods to assess infants' neurodevelopment.

**Trial registration:** NCT01245296, NCT01581489, NCT02222805, NCT01620008, IRCT201702066807N19, and NCT02727517

## Highlights

- Delayed cord clamping may improve the long-term neurological outcome of infants;
- Delayed cord clamping significantly improves the infants' domains communication and personal-social assessed through Ages and Stages Questionnaire (ASQ) score at 12 months of age;
- The ASQ personal social domain explored at 4 months seems to be significantly lower in the DCC group than in the ECC group.

## Introduction

The third stage of labor has always been of great interest both for obstetricians and neonatologists. This critical time, between the baby's birth to the placenta and membranes' expulsion, could be accompanied by a considerable maternal blood loss. Nearly 125,000 women die annually across the world from excessive bleeding within the first 24 h after delivery, also known as primary post-partum hemorrhage (PPH) (1). Concerned by this problem, obstetricians abandoned the “hands-off” approach to the third stage of labor in favor of active management. This management is a package of interventions that included: –the routine administration of a prophylactic uterotonic drug at different times around the baby's birth, –early cord clamping (ECC) and cutting (before, during, or immediately after the administration of the uterotonic); and–controlled cord traction to deliver the placenta. A recent Cochrane review (2) including 8 studies for a total of 8,892 women concluded that the active management could effectively reduce the rate of PPH at the expense of a decreased birth weight in newborns. The phenomenon of placental transfusion was well-demonstrated in 1969. This study (3) showed an increasingly rapid redistribution of the blood volume between the placenta and the newborn according to the different cord clamping times. When the cord was clamped at 15 s, 73% of the blood volume was in the infant and 27% in the placenta, at 60 s 80% was in the infant and 20% in the placenta, and at 180 s 87% in the infant, while only 13% remained in the placenta. That is why ECC, which is among the main components of the third stage of labor's active approach, became questionable. Recently, a Cochrane review showed that, compared with ECC, delayed cord clamping (DCC) might increase birthweight, the mean Hemoglobin concentration in infants at 24 to 48 h, as well as their iron stores at 3 to 6 months (4). Besides these benefits, it has been suggested that DCC might also improve the infants' brain development (4). Several articles have recently been published claiming an effect of the timing of cord clamping on neonatal cerebral development. Understanding DCC's influence on term infants' neurodevelopmental outcomes is of high interest since it could impact the 140 million babies born each year globally. Therefore, the present review aims to evaluate DCC's effectiveness in improving the infants' neurodevelopment during the long term (≥4 months). The Ages and Stages Questionnaire (ASQ) is a parent-completed child developmental-behavioral screening tool, which consists of a series of age-related questionnaires, ranging from 2 to 60 months (5). Specifically, the present paper primarily aimed to assess DCC's effect on ASQ total score (during long-term infants' follow-up ≥4 months). A secondary purpose was to assess DCC's effect on ASQ domains (during long-term infants' follow-up ≥4 months) and on other methods used to evaluate the infants' neurodevelopment.

## Methods

### Search Strategy

According to the PICO framework, the specific study aim was written as previously described (6) (for the specific PICO questions, see Supplementary Table 1). The research protocol was designed a priori, methods for searching the literature were defined, and then the articles were examined for data extraction and analysis. Searches were performed in Pubmed/MEDLINE, Scopus, ClinicalTrials.gov, and the Cochrane Central Register of Controlled Trials. The PICO framework was used to develop the literature search strategy. The following search terms were used: (“cord clamping” OR “delayed cord clamping” OR “early cord clamping” OR “cord clamp”) AND (“neurodevelopment” OR “myelin content” OR “ages and stages questionnaires” OR “ASQ” OR “Neurodevelopmental testing” OR “Mullen Scales of Early Learning” OR “mullen”) from the inception of each database until November 2nd, 2020 (for details about the queries see Supplementary Table 2). No geographical restrictions were applied.

### Study Selection

Selection criteria included randomized controlled trials (RCTs) of term singleton gestations randomized to receive DCC, as defined by the original trial. Trials in multiple pregnancies, pre-term delivery, non-randomized studies (such as cohort studies, case reports, reviews, or letters to the editor), and articles in languages other than English were excluded.

### Data Extraction and Risk of Bias Assessment

The risk of bias in each included study was assessed by using the criteria outlined in The Cochrane Collaboration's tool for assessing the risk of bias in randomized trials (7). Seven domains related to the risk of bias were assessed in each trial included, since there is evidence that these issues are associated with biased estimates of treatment effect: (1) sequence generation (selection bias); (2) allocation sequence concealment (selection bias); (3) blinding of participants and personnel (performance bias); (4) blinding of outcome assessment (detection bias); (5) incomplete outcome data (attrition bias); (6) selective reporting (reporting bias); and (7) other potential sources of bias. Review authors' judgments were categorized as “low risk,” “high risk,” or “unclear risk” of bias.

### Data Collection

The following information was collected in a predetermined form: year of publication, study time-frame, study location, study type, study protocol identifier, type of method used to assess infant and child neurodevelopment, study conclusions, mother and pregnancy characteristics (age, parity, previous cesarean sections, BMI, and gestational age), fetal characteristics (fetal weight, small for gestational age, etc.). The primary outcome was the total ASQ score (during long-term infants' follow-up ≥4 months) in terms of the mean difference between the ECC and DCC groups. The secondary outcomes gave scores on five domains (communication, gross motor, fine motor, problem-solving ability, and personal social functioning). Data from each eligible study were extracted without modification of original data and onto custom-made data collection forms.

### Data Analysis

In this meta-analysis, the *p* < 0.05 was defined as statistically significant. Where possible, a summary statistic was also calculated (mean differences). Furthermore, funnel plots assessed publication bias, and where suitable, a statistical test was performed to assess if the association between estimated intervention effects and the measure of study size occurred by chance or not (8–12). The between-study heterogeneity was evaluated by the I-square index and the Cochran *Q*-tests and, respectively, I-square >50% or Q statistic *p* < 0.10 implicated the presence of statistically remarkable heterogeneity (13). A random-effects model was then used to compute pooled estimates if there was a significant heterogeneity; otherwise, a fixed-effects model was used. The primary and secondary outcome was to describe the mean difference (MD) (with 95% CI) of ASQ score and its domains between DCC study groups and ECC control groups. Where feasible, a sensitivity analysis was planned to check the robustness of the pooled effects by removing each study, one by one. Two authors (SX, AL) independently assessed electronic search, eligibility of the studies, inclusion criteria, risk of bias, data extraction. Disagreements were resolved by discussion. This meta-analysis was performed following the PRISMA (Preferred Reporting Items for Systematic Reviews and Meta-analyses) criteria (14). No ethical approval was required since this meta-analysis involves only publicly available anonymous data. The statistical program R (version 3.6.3; R Core Team−2020. R: A language and environment for statistical computing. R Foundation for Statistical Computing, Vienna, Austria–URL https://www.R-project.org/) was used for analysis and graphs.

## Results

### Study Selection and Study Characteristics

A total of 460 records were screened; after removal of duplicates, 412 records were considered. Among these, 374 were excluded because they were irrelevant or no full text was available. Therefore, 38 full-text articles were assessed for eligibility. However, 30 did not meet the inclusion criteria (17 not pertinent, 1 not RCT, 2 protocols without results, and 10 reviews) (Supplementary Material 1). Eventually, 8 studies were included in the qualitative synthesis and 4 of these in the meta-analysis.

**Figure 1:**
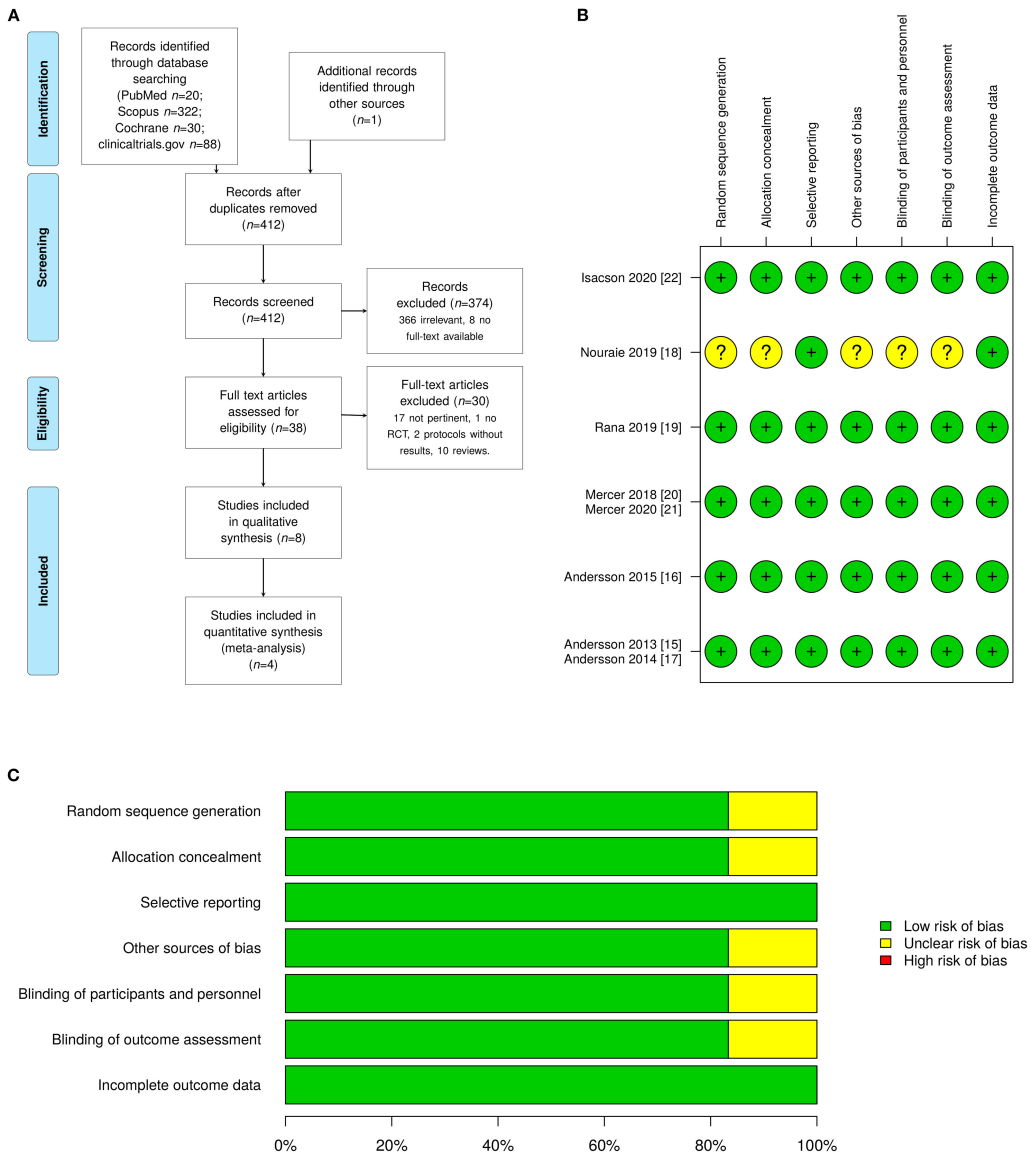
**(A)** The PRISMA flow diagram showing the literature search and selection. **(B)** Study quality summary, which includes our judgements about each risk-of-bias item for each included study [colors legend: green (+) as low risk of bias (high quality), yellow (?) as unclear, and red (–) as high risk of bias (low quality)]. **(C)** Methodological quality score summary shown as percentage of all included 6 trials (denominator) published in 8 articles.

Eight articles on long-term neurological effects in infants receiving DCC at birth met the inclusion criteria and were therefore identified as relevant (Figure 1A). All papers included healthy term pregnancies and compared two different approaches of cord clamping: delayed (90–180 s) vs. early (<60 s). The articles correspond with 6 different clinical trial protocols, two of which involve the same population (15, 16) (Supplementary Table 3). Four trials (15, 17–19) used the same tool for neurological assessment, the Ages and Questionnaire series, and on this basis, they were included in the present meta-analysis. Four trials (16, 20–22) explored the neurodevelopmental outcome by using other methods. The only trial (16) that analyzed children at 4 years of age used the Wechsler Pre-school and Primary Scale of Intelligence (WPPSI-III) test, which allows for cognitive assessment in children at 4 to 7 years of age. Another trial (22), carried out in a low-income country, used the Infant and Young Child Development (IYCD) method; this is a standardized test for estimating cognitive development in children up to 3 years of age across different cultures. The Mullen Scales of Early Learning was used at 4 and 12 months follow-up by Mercer et al.; this instrument is a standardized and population-normed tool for assessing fine and gross motor control, visual reception, and expressive and receptive language in children up to 5 years (20, 21). In the two studies of Mercer et al. the infant neurological evaluation was implemented with a novel MRI technique (mcDESPOT, multicomponent-Driven Equilibrium Single-Pulse Observation of T1 and T2) that was able to determine the myelin content in the brain (20, 21). The characteristics of the RCTs deemed relevant for this systematic review are summarized in Table 1. Primary and secondary outcomes are also summarized in Table 1. All studies, except for one (17), found a favorable association between the DCC and infant neurodevelopment, either in certain domains of neurological skills or in the general evaluation. Moreover, when mc-DESPOT MRI was used, an increased myelin content in areas important for early life function was observed.

**Table 1:** Description of the included studies in the qualitative analysis.

**References**	**Country**	**Inclusion criteria**	**Comparison**	**Number of participants (DCC vs. ECC)**	**Neurodevelopmental outcomes**	**Study conclusions**
Andersson et al. (15)	Sweden	Healthy, term pregnants, expected vaginal delivery	DCC (≥180 s) vs ECC (*≤* 10 s)	185 vs. 180	Neurodevelopment assessed by ASQ (*) at 4 months of age	DCC did not affect overall neurodevelopment, but may have an impact on specific neurodevelopmental domains (problem solving)
Andersson et al. (17)	Sweden	Healthy, term pregnants, expected vaginal delivery	DCC (≥180 s) vs ECC (*≤* 10 s)	172 vs. 168	Neurodevelopment assessed by ASQ at 12 months	DCC did not affect neurodevelopment at age 12 months
Andersson et al. (16)	Sweden	Healthy, term pregnants, expected vaginal delivery	DCC (≥180 s) vs ECC (*≤* 10 s)	112 vs. 104	Neurodevelopment assessed by WPPSI-III (†) at 48–51 months of age. Assessment of fine motor skills (‡) (through Movement ABC), psychomotor development (through ASQ) and behavior (§) (through SDQ)	DCC improved scores in the fine motor and social domains
Mercer et al. (20)	USA	Term, uncomplicated pregnancies	DCC (90–120 s) vs. ECC (<60 s)	23 vs. 21	MRI during natural non sedated sleep at 4 months (measure of brain myelin content). Neurodevelopmental testing with Mullen Scales of Early Learnin (¶) at 4 months	DCC improved brain myelin in areas important for early life functional development
Mercer et al. (21)	USA	Term, uncomplicated pregnancies	DCC (90–120 s) vs. ECC (<60 s)	21 vs. 20	MRI during natural non sedated sleep at 12 months Neurodevelopmental testing with Mullen Scales of Early Learning at 12 months	DCC increased myelin content in important brain regions involved in motor function, visual/spatial, and sensory processing
Nouraie et al. (18)	Iran	Term, uncomplicated pregnancies	DCC (90–120 s) vs. ECC (<60 s)	200 vs. 200	Neurodevelopment assessed by ASQ at 4 months of age	DCC has no effect on infant development, except for problem solving skills
Rana et al. (19)	Nepal	Healthy, term pregnants, expected vaginal delivery	DCC (≥180 s) vs. ECC (*≤* 10 s)	173 vs. 159	Neurodevelopment assessed by ASQ at 12 months of age	DCC was associated with an improvement of the overall neurodevelopment at 12 months
Isacson et al. (22)	Nepal	Healthy, late preterm and term pregnants, expected vaginal delivery	Resuscitation while DCC (≥180 s) vs. resuscitation after ECC (<60 s)	84 vs. 54	Total score for the IYCD (#) at 2 years Total scores for the subdomains: motor, language, cognitive, and socio-emotional at 2 years	Resuscitation while DCC improved neurodevelopment in infants at 2 years of age

(*) *The Ages and Stages Questionnaire (ASQ) is a series of age-related questionnaires, ranging from 2 to 60 months, that are completed by parents. Each questionnaire comprises 3 sections: a brief section of demographic items, 30 questions covering the infants or childs' development in 5 different domains: communication, gross motor, fine motor, problem solving, and personal-social, and seven open-ended questions in 5 different domains*.

(†) *The Wechsler Pre-school and Primary Scale of Intelligence, WPPSI-III, is a test that allows to assess cognitive function in children at 4–7 years of age. WPPSI-III composite scores are defined by verbal IQ, performance IQ, processing speed quotient, and general language composite*.

(‡) *The Movement Assessment Battery for Children (Second Edition) assesses fine motor skills and includes 3 subsets: time for posting coins into a slot, time for bead threading, and drawing within a bicycle trail*.

(§) *The Strengths and Difficulties Questionnaire (SDQ), assesses the behavior and is directed to children at 3 to 4 years. This test includes 5 subscales: emotional difficulties, conduct difficulties, hyperactivity difficulties, peer problem, and prosocial score*.

(¶) *Mullen Scales of Early Learning is a standardized and population normed tool for assessing fine and gross motor control, visual reception, and expressive and receptive language for children up to 5 years*.

(#) *The Infant and Young Child Development (IYCD) provides a standardized method to estimate development, at the population level, for children up to 3 years of age across cultures. The IYCD contains four subdomains: gross motor, language, cognitive, and socio-emotional*.

### Quality and Risk of Bias Assessment

All the included studies except one were single blinded RCTs (15–17, 19–22). In the remaining RCT the information about blinding was not completely clear (18). However, as clearly stated in many studies (15–17, 22), the mother could not be blinded as well as the personal attending the birth (15–17, 19–22). The methodological quality of the trials was addressed by adopting the Cochrane collaboration's tool for assessing the risk of bias: Figures 1B,C, respectively, report the detailed scores and the overall summary.

### Publication Bias

For the studies included in the quantitative synthesis, publication bias was also assessed. Due to the limited number of included studies, only the funnel plot was used in the quantitative synthesis to assess the publication bias, as shown in Supplementary Figure 1.

### ASQ Assessment

**Figure 2:**
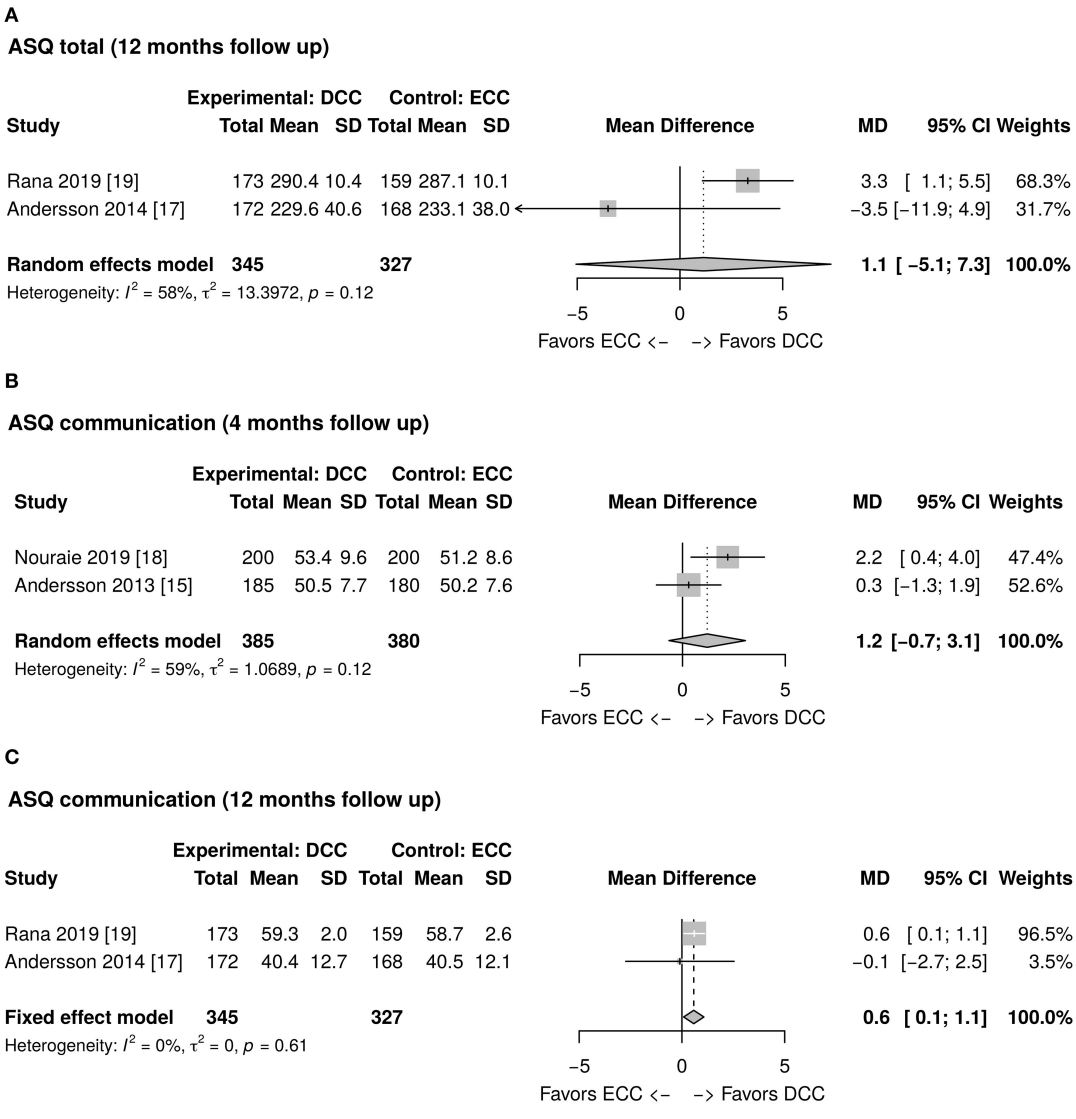
**(A)** Forest plot showing mean difference of the Ages and Stages Questionnaire (ASQ) total score at 12 months follow up between delayed cord clamping (DCC) and early cord clamping (ECC) groups. **(B)** Forest plot showing mean difference of the ASQ communication domain score at 4 months follow up between DCC and ECC groups. **(C)** Forest plot showing mean difference of the ASQ communication domain score at 12 months follow up between DCC and ECC groups.

**Figure 3:**
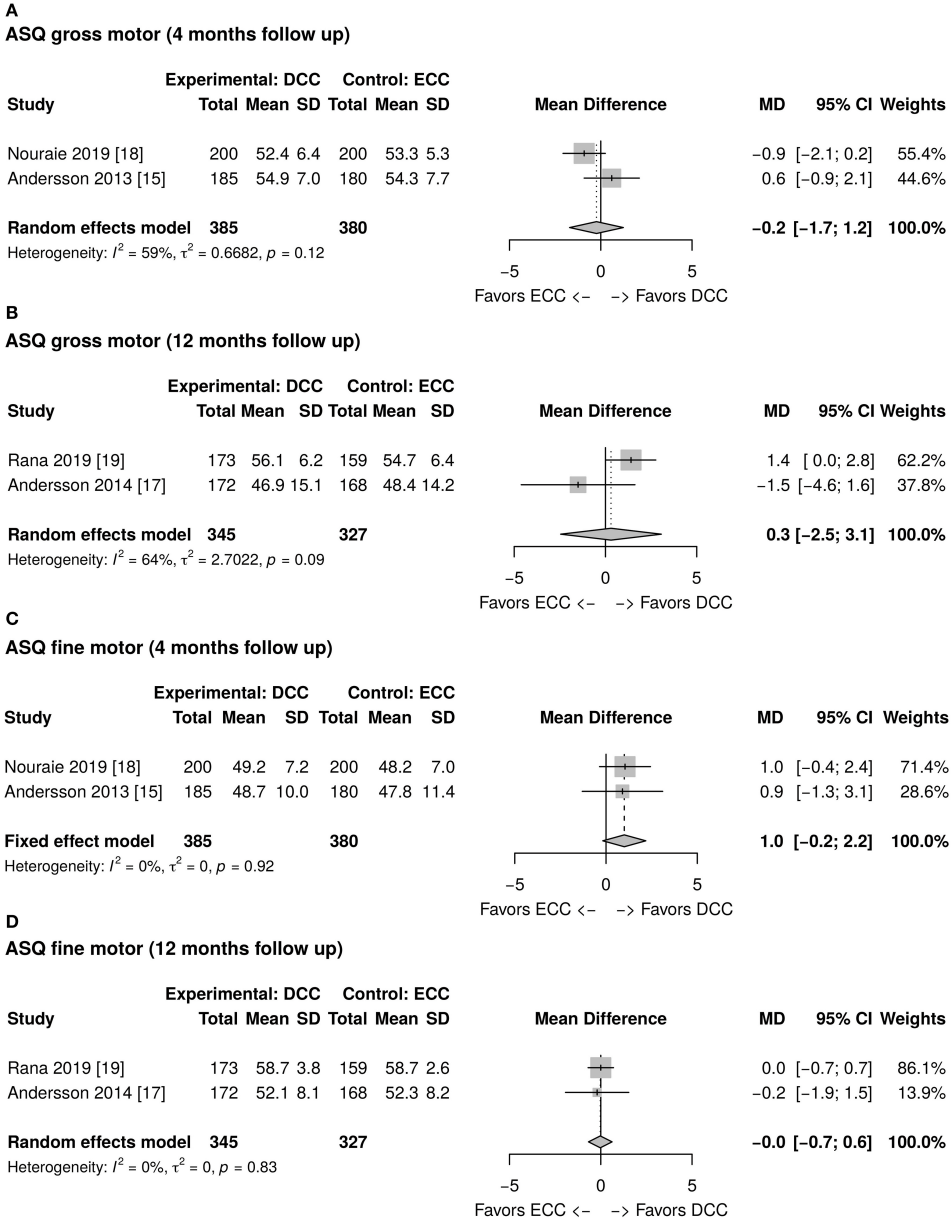
**(A)** Forest plot showing mean difference of the Ages and Stages Questionnaire (ASQ) gross motor domain score at 4 months follow up between delayed cord clamping (DCC) and early cord clamping (ECC) groups. **(B)** Forest plot showing mean difference of the ASQ gross motor domain score at 12 months follow up between DCC and ECC groups. **(C)** Forest plot showing mean difference of the ASQ fine motor domain score at 4 months follow up between DCC and ECC groups. **(D)** Forest plot showing mean difference of the ASQ fine motor domain score at 12 months follow up between DCC and ECC groups.

**Figure 4:**
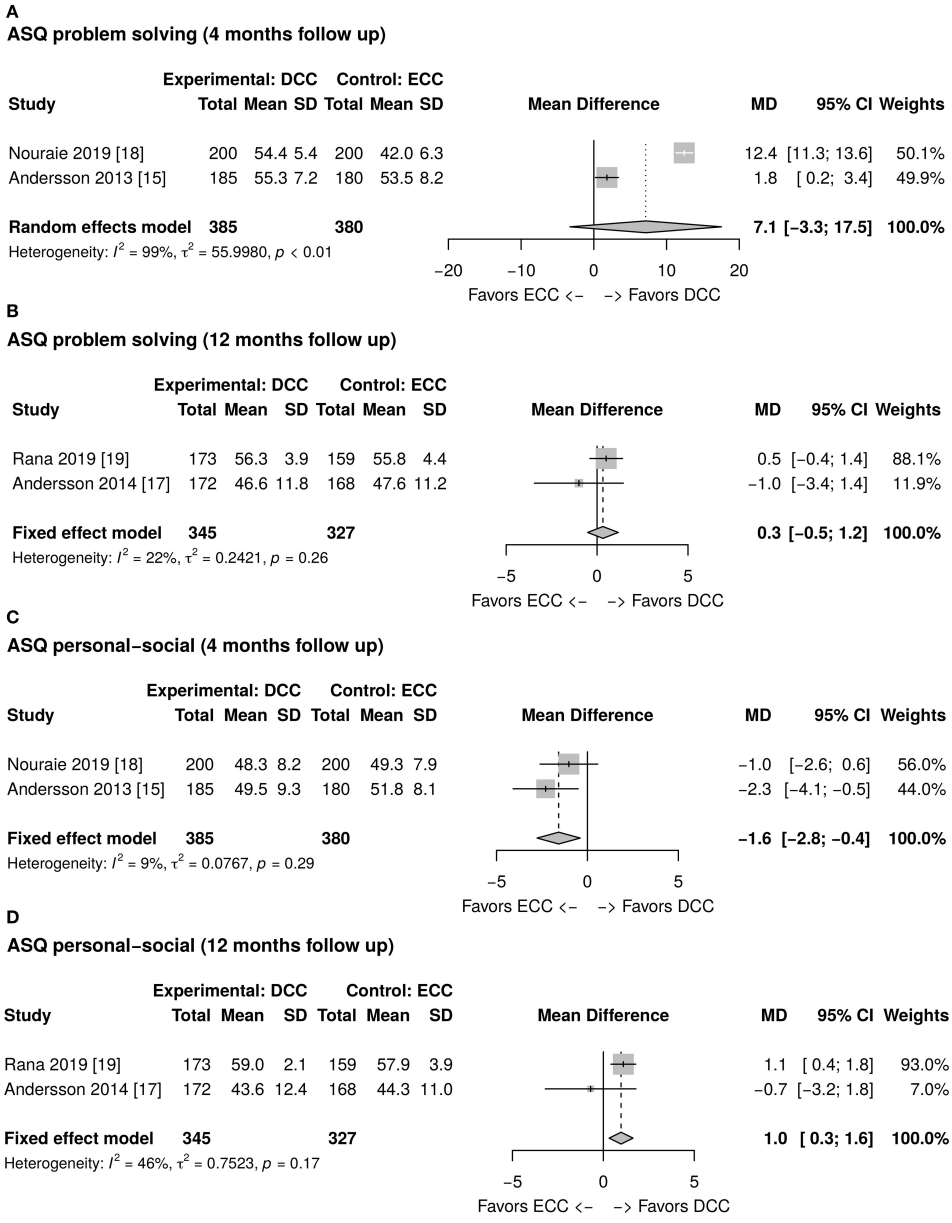
**(A)** Forest plot showing mean difference of the Ages and Stages Questionnaire (ASQ) problem solving domain score at 4 months follow up between delayed cord clamping (DCC) and early cord clamping (ECC) groups. **(B)** Forest plot showing mean difference of the ASQ problem solving domain score at 12 months follow up between DCC and ECC groups. **(C)** Forest plot showing mean difference of the ASQ personal-social domain score at 4 months follow up between DCC and ECC groups. **(D)** Forest plot showing mean difference of the ASQ personal-social domain score at 12 months follow up between DCC and ECC groups.

As reported in Figure 2A, the total ASQ assessment score evaluated at 12 months of life is higher in the DCC group than in the ECC group. However, the mean difference is not statistically significant (1.1; CI 95: −5.1; 7.3). The ASQ five domains assessment results at 4 and 12 months of age have been meta-analyzed and are reported in Figures 2B,C, 3A–D, 4A–D. Figure 2C shows a favorable score for DCC in the communication domain at 12 months of age with a mean difference between the groups of 0.6 (CI 95: 0.1; 1.1). The personal-social domain at 4 months of age was higher in the ECC group with a mean difference of −1.6 (CI 95: −2.8; −0.4), while the same domain explored at 12 months turned out to be higher in the DCC group (mean difference 1.0 CI 95: 0.3; 1.6) (see Figures 4C,D).

## Comment

### Main Findings

This meta-analysis of 4 articles from 3 randomized clinical trials showed that a policy of delaying cord clamping improves significantly the infants' domains of communication and personal-social assessed through ASQ at 12 months of age. Surprisingly, the ASQ personal-social domain explored at 4 months seems to be significantly lower in the DCC group than in the ECC group.

### Comparison With Existing Literature and Implication

So far, the literature in term infants has mainly focused on the improvement of hematological parameters in the first months of life after the blood transfer occurred from the placenta toward the newborn while the cord was still intact at birth. The present paper draws attention to a relatively new issue: the long-term effect of placental transfusion on early infancy neurodevelopment. The results of this meta-analysis suggest a positive impact of the extra blood volume received by infants in the first minutes after birth on cognitive and behavioral skills, evaluated through ASQ at 12 months of life. However, two studies by Mercer et al. (20, 21), not included in the quantitative analysis, measured with a novel MRI technique the myelin content in infants exposed to placental transfusion at 4 and 12 months, respectively. These studies (20, 21) found not only that infants whose cord was clamped and cut after 5 min had an enhanced myelin formation in their brain, but also that this higher content of myelin was likely due to their larger iron stores, facilitated by placental transfusion. In the first two post-natal years, white matter develops rapidly. The synthesis and maintenance of myelin, the most critical function of oligodendrocytes, requires high iron levels to deal with the enzymatic and metabolic needs. What emerges from the studies carried out by Mercer et al. is that a simple procedure at birth, like waiting a few minutes before umbilical cord clamping, could enrich infants' iron stores, critical for the high energy demands of oligodendrocytes. It might be hypothesized that this challenging observation explains why the domains communication and personal-social of infants treated with DCC at birth and assessed through ASQ at 12 months of age resulted improved. Iron deficiency especially targets neonates and young children with an estimated prevalence of 42% (23) and 80% (24) in high-income but also in low-income countries. Two trials have been conducted in low-income countries: Nepal and Iran. DCC could be considered the first step to reduce anemia in the first years of life, especially in low-income settings with a high prevalence of iron deficiency anemia, where the post-natal nutrition could not be adequate for the infants' energy requirements. In such conditions, placental transfusion's long-term effect could be significant for subsequent brain development. Interestingly, Isacson et al. (22) performed DCC in neonates necessitating resuscitation because of birth asphyxia (i.e., not breathing despite thorough drying and additional stimulation). This trial achieved promising results since the neurodevelopment at 2 years of age appeared to be better in those babies resuscitated with an intact cord than in babies resuscitated immediately after ECC.

### Strengths and Limitations

To our knowledge, this is the first meta-analysis that identifies a significant association between the DCC and the pre-school age developmental outcomes (personal-social and communication) in a multiethnic population of infants born vaginally at the term of gestation. However, multiple limitations should be acknowledged when interpreting this systematic review and meta-analysis findings. First, all the included studies are single-blinded, and parents were aware of the treatment in all cases. This blinding system could have influenced the scores obtained when parents were involved in compiling the questionnaires to assess their infants' neurodevelopment. However, although this is true for questionnaires like ASQ, presumably, it is not valid for the objective cerebral MRI assessment performed by Mercer et al. (20, 21). Second, due to the limited number of studies included in the quantitative synthesis, the publication bias was assessed only by funnel plots (12). However, even though most of the analysis showed no bias, a possible bias was found in Supplementary Figure 1H, which considers ASQ problem-solving domain score. In this case, the funnel plot was found to have asymmetry. Publication bias is not the only cause of funnel plot asymmetry. Other possible causes could be: location biases, poor methodological design, inadequate analysis, or fraud (12). In particular, Supplementary Figure 1H is presumably the result of the higher than the average mean difference shown in the study of Nouraie et al. (18). Third, language bias may have been introduced by excluding non-English language studies; however, no pertinent items in non-English language were found. Fourth, the inclusion of only full-text articles might lead to a full-text bias that might influence the findings of this meta-analysis (25). Nonetheless, the full text of each pertinent article was retrieved. Indeed, among the 8 items where the full text was not available, 3 were congress-abstracts, 3 protocols and 2 commentaries. Fifth, another limitation of the study can be the number of comparisons in the secondary outcomes that could raise the multiplicity issue. However, although exploratory, our approach during the study planning focused on a well-defined primary outcome, reducing the secondary outcomes comparisons to well-established comparisons made in the original articles. A last but not less relevant issue is that the type of developmental score used in these studies could have been inadequate for the aims considered. For example, the ASQ, when compared with the well-accepted Bayley-III scale, has been recognized to have some limitations (26). Generally, it would have been preferable to assess DCC's efficacy with validated developmental scores that are independent from the parent assessment. The discrepancy in the infants' personal-social assessments, which are lower in the DCC vs. ECC when looked at 4 months, but significantly higher in the DCC group at 12 months, raises concerns about early screening effectiveness in predicting long-term outcomes. Voss et al. showed that cognitive-developmental prognosis in 129 pre-term babies was correct in only 49% of cases, and this percentage increased to 70% at the age of 3–4 years (27). Similarly, Hack et al. demonstrated that a subnormal Mental developmental Index score at 20 months of corrected age in 330 pre-term infants was poorly predictive of their cognitive function at 8 years (28). Interestingly, both authors concluded that early testing is unreliable for later outcomes, except for the most severe neurodevelopmental impairment. These results emphasize the need to assess the children's neurodevelopment at school age. Furthermore, another matter of concern is the accuracy of the ASQ tool in identifying developmentally delayed children. Despite being a low-cost screening tool with a short completion time, easily performed in a home setting, ASQ has not been proven to be an accurate measure for screening children at risk of neurodevelopmental disorders. By contrast, Lamsal et al. demonstrated that the ASQ was effective at identifying children with a high risk of neurodevelopmental delay when a 1 standard deviation cut-off was used at 24 months (29).

### Generalizability

The generalization of our findings might be impaired because of the low number of clinical trials on this topic, which does not allow to draw definitive conclusions. It should be recommended to perform more clinical trials to strengthen the current findings. Our meta-analysis includes very different populations. By comparing participants living in low- and high-income countries, multiple factors of diversity, such as socio-economic status, environmental influences, pre-, and post-natal care, need to be considered. The fact that results coming from different populations are going in the same direction is, in some way, reassuring and may confirm a potential benefit of the DCC. Last, generalizability might be impaired by the lack of standardizing the timing of cord clamping in routine clinical practice. Time disparities in cord clamping do exist, reflecting the variability in adherence to DCC recommendations among health care workers. DCC policy seems challenging to apply, especially when the newborn is perceived to require assistance, from simple, thorough drying to additional stimulation and ventilation.

## Conclusions

This systematic review and meta-analysis of randomized clinical trials show that DCC has no impact on ASQ total score during long-term infants' follow-up. However, some neurological skills, assessed by ASQ subdomains, were significantly modified by this non-interventional and cost-effective approach. To confirm these observations, further research should be recommended, preferably with randomized controlled trials using the same method for neurological skills assessment in infants, to allow outcome consistency within the literature and in association with validated developmental scores independent of the parent assessment.

## Key Message

Delayed cord clamping may improve the long-term neurological outcome of infants.

## Condensation

This review of randomized clinical trials suggests that delayed cord clamping, a simple, non-interventional and cost-effective approach, may improve the long-term neurological outcome of infants.

## Data Availability Statement

The original contributions presented in the study are included in the article/Supplementary Material, further inquiries can be directed to the corresponding author/s.

## Author Contributions

SX, LX, GB, LD, and AL: substantial contributions to conception, design or acquisition of data or to analysis, interpretation of data, and drafting the article or revising it critically for important intellectual content. All authors have read and approved the final manuscript.

## Conflict of Interest

The authors declare that the research was conducted in the absence of any commercial or financial relationships that could be construed as a potential conflict of interest.

## Supplementary Material

Supplementary Figure 1 Funnel plots. **(A)** The Ages and Stages Questionnaire (ASQ) total score at 12 months follow up. **(B)** The ASQ communication domain score at 4 months follow. **(C)** The ASQ communication domain score at 12 months follow. **(D)** The ASQ gross motor domain score at 4 months follow. **(E)** The ASQ gross motor domain score at 12 months follow. **(F)** The ASQ fine motor domain score at 4 months follow. **(G)** The ASQ fine motor domain score at 12 months follow. **(H)** The ASQ problem solving domain score at 4 months follow. **(I)** The ASQ problem solving domain score at 12 months follow. **(J)** The ASQ personal-social domain score at 4 months follow. **(K)** The ASQ personal-social domain score at 12 months follow.

Supplementary Table 1 Summary of the systematic review aims. The primary aim was formulated on the basis of PICO framework. ASQ, Ages and stages questionnaire; DCC, delayed cord clamping; ECC, early cord clamping; PICO, patients, intervention, comparator, and outcome.

Supplementary Table 2 Summary of database queries.

Supplementary Table 3 Characteristics of the included trials. (^*^) This trial (NCT01581489) was a follow up of the same cohort of NCT01245296.

Supplementary Material 1 Included and excluded studies.

## Abbreviations

ASQ: Ages and Stages Questionnaire
CI: Confidence Interval
DCC: Delayed Cord Clamping
ECC: Early Cord Clamping
IQ: Intelligence Quotient
IYCD: Infant and Young Child Development
mcDESPOT: multicomponent-Driven Equilibrium Single-Pulse Observation of T1 and T2
MD: Mean Difference
MRI: Magnetic Resonance Imaging
PICO: Patients, Intervention, Comparator, and Outcome
PPH: Post-Partum Hemorrhage
PRISMA: Preferred Reporting Items for Systematic Reviews and Meta-Analyses
RCT: Randomized Controlled Trial
SDQ: Strengths and Difficulties Questionnaire
WPPSI-III: Wechsler Pre-school and Primary Scale of Intelligence III.
