# Supplementary figures and images for: Does Delayed Cord Clamping Improve Long-Term (≥4 Months) Neurodevelopment in Term Babies? A Systematic Review and a Meta-Analysis of Randomized Clinical Trials
Source: Front Pediatr. 2021 Apr 12;9:651410. doi: 10.3389/fped.2021.651410 (PMC8071880; doi:10.3389/fped.2021.651410)

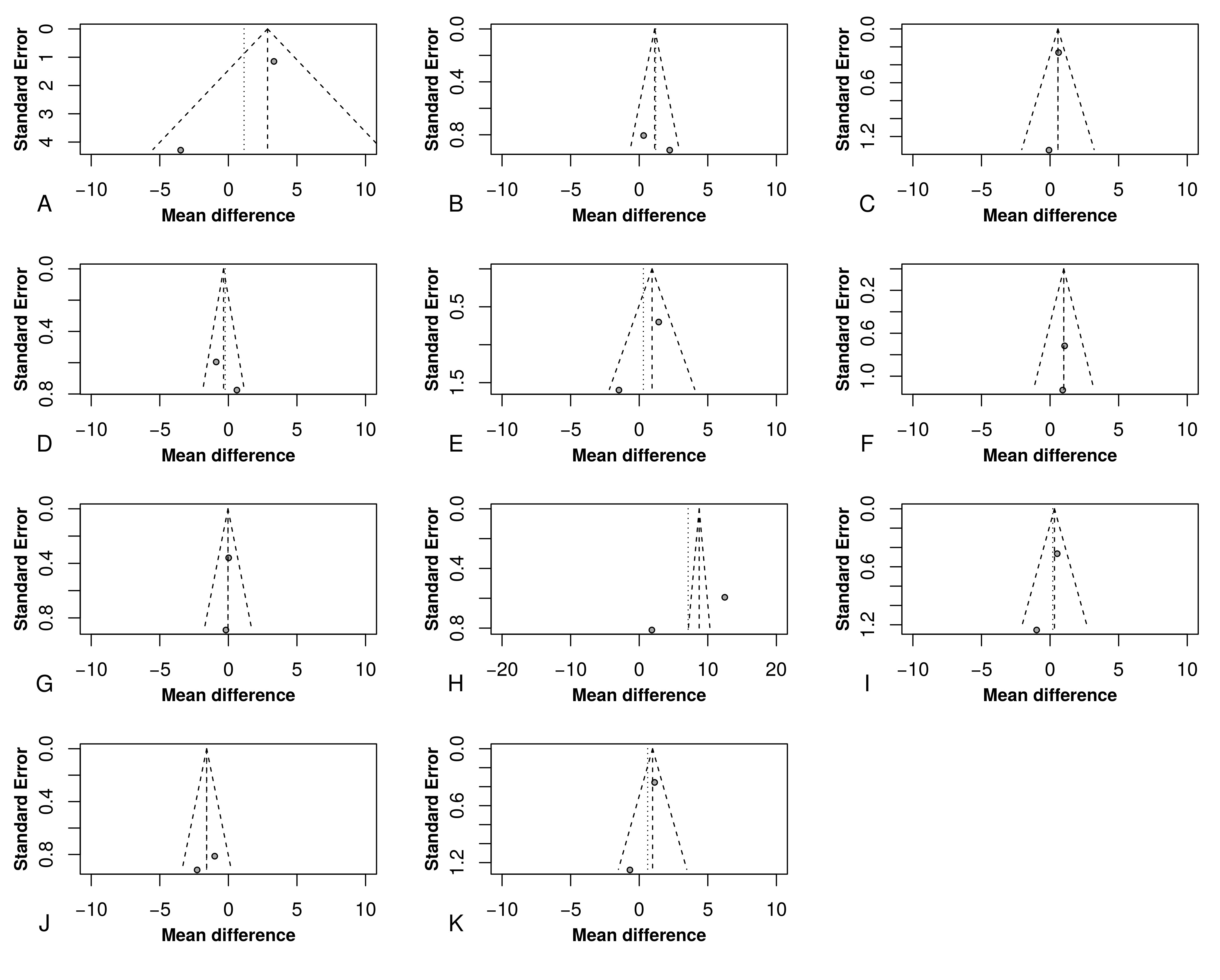

Supplement: Supplementary Figure 1 — Funnel plots. (A) The Ages and Stages Questionnaire (ASQ) total score at 12 months follow up. (B) The ASQ communication domain score at 4 months follow. (C) The ASQ communication domain score at 12 months follow. (D) The ASQ gross motor domain score at 4 months follow. (E) The ASQ gross motor domain score at 12 months follow. (F) The ASQ fine motor domain score at 4 months follow. (G) The ASQ fine motor domain score at 12 months follow. (H) The ASQ problem solving domain score at 4 months follow. (I) The ASQ problem solving domain score at 12 months follow. (J) The ASQ personal-social domain score at 4 months follow. (K) The ASQ personal-social domain score at 12 months follow. [file Image_1.JPEG]
