# Supplementary material for: Does Delayed Cord Clamping Improve Long-Term (≥4 Months) Neurodevelopment in Term Babies? A Systematic Review and a Meta-Analysis of Randomized Clinical Trials
Source: Front Pediatr. 2021 Apr 12;9:651410. doi: 10.3389/fped.2021.651410 (PMC8071880; doi:10.3389/fped.2021.651410)
Supplement: Supplementary Table 1 — Summary of the systematic review aims. The primary aim was formulated on the basis of PICO framework. ASQ, Ages and stages questionnaire; DCC, delayed cord clamping; ECC, early cord clamping; PICO, patients, intervention, comparator, and outcome. [file Table_1.DOCX]

**Supplemental Table 1**- Summary of the systematic review aims. The primary aim was formulated on the basis of PICO framework.

| N. | **Primary question** |
| --- | --- |
| 1 | What is the effect in terms of infant and child neurodevelopment (during long follow up ≥4 months), assessed as ASQ total score, of DCC in comparison to ECC at the time of third stage of labor? |
|  | **Secondary questions** |
| 2 | What is the effect in terms of infant and child neurodevelopment (during long follow up ≥4 months), assessed as ASQ sub-domain scores, of DCC in comparison to ECC at the time of third stage of labor? |
| 3 | What is the effect in terms of infant and child neurodevelopment (during long follow up ≥4 months), assessed with other methods than ASQ score, of DCC in comparison to ECC at the time of third stage of labor? |

Acronyms: ASQ = Ages and Stages Questionnaire; DCC = delayed cord clamping; ECC = early cord clamping; PICO = Patients, Intervention, Comparator, and Outcome.

**Supplemental Table 2**- Summary of database queries.

| **Database** | **Query** | **Date** | **Number of items** |
| --- | --- | --- | --- |
| PubMed | ("cord clamping" OR "delayed cord clamping" OR "early cord clamping" OR "cord clamp") AND ("neurodevelopment" OR "myelin content" OR "ages and stages questionnaires" OR "ASQ" OR "Neurodevelopmental testing" OR "Mullen Scales of Early Learning" OR "mullen") | 02.11.2020 | 20 |
| Scopus | ( "cord clamping" OR "delayed cord clamping" OR "early cord clamping" OR "cord clamp" ) AND ( "neurodevelopment" OR "myelin content" OR "ages and stages questionnaires" OR "ASQ" OR "Neurodevelopmental testing" OR "Mullen Scales of Early Learning" OR "mullen" ) | 02.11.2020 | 322 |
| Cochrane Central Register of Controlled Trials | ("cord clamping" OR "delayed cord clamping" OR "early cord clamping" OR "cord clamp") AND ("neurodevelopment" OR "myelin content" OR "ages and stages questionnaires" OR "ASQ" OR "Neurodevelopmental testing" OR "Mullen Scales of Early Learning" OR "mullen") in All Text - (Word variations have been searched) | 02.11.2020 | 30 |
| clinicaltrials.gov | ("cord clamping" OR "delayed cord clamping" OR "cord clamp") [limited to Interventional Studies and Child] | 02.11.2020 | 88 |

**Supplemental Table 3**- Characteristics of the included trials.

| Protocol number | First author and year of publication | Location | Country | Enrollment |
| --- | --- | --- | --- | --- |
| NCT01245296 (*) | Andersson 2013 [15] and Andersson 2014 [17] | Hospital of Halland, Halmstad | Sweden | April 2008-May 2009 |
| NCT01581489 (*) | Andersson 2015 [16] | Hospital of Halland, Halmstad | Sweden | April 2008-May 2009 |
| NCT02222805 | Rana 2019 [19] | Paropakar Maternity and Women’s Hospital, Kathmandu | Nepal | October 2, 2014-November 21, 2014 |
| NCT01620008 | Mercer 2018 [20] and Mercer 2020 [21] | Women and Infants Hospital of Rhode Island and Brown University (Providence, Rhode Island) | USA | July 2012-November 2015 |
| IRCT201702066807N19 | Nouraie 2019 [18] | Isfahan University of Medical Sciences and Health Services, Isfahan | Iran | 2016 |
| NCT02727517 | Isacson 2020 [22] | Paropakar Maternity and Women's, Kathmandu | Nepal | April 20 to August 27, 2016 |

(*) This trial (NCT01581489) was a follow up of the same cohort of NCT01245296.
