# Supplementary material for: Does Delayed Cord Clamping Improve Long-Term (≥4 Months) Neurodevelopment in Term Babies? A Systematic Review and a Meta-Analysis of Randomized Clinical Trials
Source: Front Pediatr. 2021 Apr 12;9:651410. doi: 10.3389/fped.2021.651410 (PMC8071880; doi:10.3389/fped.2021.651410)
Supplement: Supplementary Material 1 — Included and excluded studies. [file Data_Sheet_1.PDF]

Supplemental List 1

INCLUDED STUDIES

Studies that fulfilled the requirements.

[1] Rana N, Kc A, Målqvist M, Subedi K, Andersson O. Effect of Delayed Cord Clamping of Term Babies on Neurodevelopment at 12 Months: A Randomized Controlled Trial. Neonatology. 2019;115:36–42.

[2] Nouraie S, AMIRALII Akbari S, Vameghi R, Akbarzade Baghban A. The Effect of the Timing of Umbilical Cord Clamping on Hemoglobin Levels, Neonatal Outcomes and Developmental Status in Infants at 4 Months Old. Iran J Child Neurol. 2019;13:45–55.

[3] Mercer JS, Erickson-Owens DA, Deoni SCL, Dean Iii DC, Tucker R, Parker AB, Joelson S, Mercer EN, Collins J, Padbury JF. The Effects of Delayed Cord Clamping on 12-Month Brain Myelin Content and Neurodevelopment: A Randomized Controlled Trial. Am J Perinatol. 2020;.

[4] Andersson O, Lindquist B, Lindgren M, Stjernqvist K, Domellöf M, Hellström-Westas L. Effect of Delayed Cord Clamping on Neurodevelopment at 4 Years of Age: A Randomized Clinical Trial. JAMA Pediatr. 2015;169:631–638.

[5] Andersson O, Domellöf M, Andersson D, Hellström-Westas L. Effect of Delayed vs Early Umbilical Cord Clamping on Iron Status and Neurodevelopment at Age 12 Months: A Randomized Clinical Trial. JAMA Pediatr. 2014; 168:547–554.

[6] Mercer JS, Erickson-Owens DA, Deoni SCL, Dean DC, Collins J, Parker AB, Wang M, Joelson S, Mercer EN, Padbury JF. Effects of Delayed Cord Clamping on 4-Month Ferritin Levels, Brain Myelin Content, and Neurodevelopment: A Randomized Controlled Trial. J Pediatr. 2018;203:266–272.e2.

[7] Andersson O, Domellöf M, Andersson D, Hellström-Westas L. Effects of Delayed Cord Clamping on Neurodevelopment and Infection at Four Months of Age: A Randomised Trial. Acta Paediatr. 2013;102:525–531.

[8] Isacson M, Gurung R, Basnet O, Andersson O, Kc A. Neurodevelopmental Outcomes of a Randomised Trial of Intact Cord Resuscitation. Acta Paediatr. 2020;.

EXCLUDED STUDIES

- Not pertinent (commentary/letter without data, no considered outcomes, preterm infants) [1–17];
- No RCT [18];
- Published protocols [19, 20];
- Reviews [21–30].

[1] De Bernardo G, Giordano M, De Santis R, Castelli P, Sordino D, Trevisanuto D, Buonocore G, Perrone S. A Randomized Controlled Study of Immediate versus Delayed Umbilical Cord Clamping in Infants Born by Elective Caesarean Section. Ital J Pediatr. 2020;46:71.

[2] Hosono S, Mugishima H, Fujita H, Hosono A, Okada T, Takahashi S, Masaoka N, Yamamoto T. Blood Pressure and Urine Output during the First 120 h of Life in Infants Born at Less than 29 Weeks' Gestation Related to Umbilical Cord Milking. Arch Dis Child Fetal Neonatal Ed. 2009;94:F328–331.

[3] Yadav AK, Upadhyay A, Gothwal S, Dubey K, Mandal U, Yadav CP. Comparison of Three Types of Intervention to Enhance Placental Redistribution in Term Newborns: Randomized Control Trial. J Perinatol. 2015;35:720–724.

[4] Bradshaw LE, Pushpa-Rajah A, Dorling J, Mitchell EJ, Duley L, Cord Pilot Trial Collaborative Group. Cord Pilot Trial: Update to Randomised Trial Protocol. Trials. 2015;16:407.

[5] Tiemersma S, Heistein J, Ruijne R, Lopez G, van Lobenstein J, van Rheenen P. Delayed Cord Clamping in South African Neonates with Expected Low Birthweight: A Randomised Controlled Trial. Trop Med Int Health. 2015;20:177–183.

[6] Cavallin F, Galeazzo B, Loretelli V, Madella S, Pizzolato M, Visentin S, Trevisanuto D. Delayed Cord Clamping versus Early Cord Clamping in Elective Cesarean Section: A Randomized Controlled Trial. Neonatology. 2019; 116:252–259.

[7] Rana N, Ranneberg LJ, Målqvist M, Kc A, Andersson O. Delayed Cord Clamping Was Not Associated with an Increased Risk of Hyperbilirubinaemia on the Day of Birth or Jaundice in the First 4 Weeks. Acta Paediatr. 2020;109:71–77.

[8] Alzaree F, Elbohoty A, Abdellatif M. Early Versus Delayed Umbilical Cord Clamping on Physiologic Anemia of the Term Newborn Infant. Open Access Maced J Med Sci. 2018;6:1399–1404.

[9] Capitulo KL, Rohan A. Toward Evidence-Based Practice:. MCN Am J Matern Nurs. 2016;41:256–258.

[10] Popat H, Robledo KP, Sebastian L, Evans N, Gill A, Kluckow M, Sinhal S, de Waal K, Tarnow-Mordi W, Osborn D. Effect of Delayed Cord Clamping on Systemic Blood Flow: A Randomized Controlled Trial. J Pediatr. 2016; 178:81–86.e2.

[11] Purisch SE, Ananth CV, Arditi B, Mauney L, Ajemian B, Heiderich A, Leone T, Gyamfi-Bannerman C. Effect of Delayed vs Immediate Umbilical Cord Clamping on Maternal Blood Loss in Term Cesarean Delivery: A Randomized Clinical Trial. JAMA. 2019;322:1869–1876.

[12] Kc A, Rana N, Målqvist M, Jarawka Ranneberg L, Subedi K, Andersson O. Effects of Delayed Umbilical Cord Clamping vs Early Clamping on Anemia in Infants at 8 and 12 Months: A Randomized Clinical Trial. JAMA Pediatr. 2017;171:264–270.

[13] Welsh S, Elwell J, Manister NN, Gildersleeve RK. Implementing Delayed Umbilical Cord Clamping in Cesarean Birth Using a Novel Method: A Pilot Study of Feasibility and Safety. J Midwifery Womens Health. 2020;65:109–118.

[14] Li Y, Zou Y, Han C, Liu X, Jiang M. Influence of Delayed Umbilical Cord Clamping on Pain during Suture of Perineal Tears: A Randomised Controlled Study. J Clin Nurs. 2020;.

[15] Agarwal S, Jaiswal V, Singh D, Jaiswal P, Garg A, Upadhyay A. Randomised Control Trial Showed That Delayed Cord Clamping and Milking Resulted in No Significant Differences in Iron Stores and Physical Growth Parameters at One Year of Age. Acta Paediatr. 2016;105:e526–e530.

[16] Berglund SK, Chmielewska AM, Domellöf M, Andersson O. Hepcidin Is a Relevant Iron Status Indicator in Infancy: Results from a Randomized Trial of Early vs. Delayed Cord Clamping. Pediatr Res. 2020;.

[17] Armstrong-Buisseret L, Powers K, Dorling J, Bradshaw L, Johnson S, Mitchell E, Duley L. Randomised Trial of Cord Clamping at Very Preterm Birth: Outcomes at 2 Years. Arch Dis Child Fetal Neonatal Ed. 2020;105:292–298.

[18] Nelin V, Kc A, Andersson O, Rana N, Målqvist M. Factors Associated with Timing of Umbilical Cord Clamping in Tertiary Hospital of Nepal. BMC Res Notes. 2018;11:89.

[19] Kc A, Målqvist M, Rana N, Ranneberg LJ, Andersson O. Effect of Timing of Umbilical Cord Clamping on Anaemia at 8 and 12 Months and Later Neurodevelopment in Late Pre-Term and Term Infants; a Facility-Based, Randomized-Controlled Trial in Nepal. BMC Pediatr. 2016;16:35.

[20] Pell LG, Bassani DG, Nyaga L, Njagi I, Wanjiku C, Thiruchselvam T, Macharia W, Minhas RS, Kitsao-Wekulo P, Lakhani A, Bhutta ZA, Armstrong R, Morris SK. Effect of Provision of an Integrated Neonatal Survival Kit and Early Cognitive Stimulation Package by Community Health Workers on Developmental Outcomes of Infants in Kwale County, Kenya: Study Protocol for a Cluster Randomized Trial. BMC Pregnancy Childbirth. 2016;16:265.

[21] Fu X, Dang D, Li S, Xu Z, Wu H. Effect of Delayed Versus Early Cord Clamping on Improving Anemia in Term Infants Aged Two Months or Older - A Meta-Analysis. Indian Pediatr. 2020;57:815–819.

[22] McDonald SJ, Middleton P, Dowswell T, Morris PS. Effect of Timing of Umbilical Cord Clamping of Term Infants on Maternal and Neonatal Outcomes. Evid Based Child Health. 2014;9:303–397.

[23] McDonald SJ, Middleton P, Dowswell T, Morris PS. Effect of Timing of Umbilical Cord Clamping of Term Infants on Maternal and Neonatal Outcomes. Cochrane Database Syst Rev. 2013;CD004074.

[24] Zhao Y, Hou R, Zhu X, Ren L, Lu H. Effects of Delayed Cord Clamping on Infants after Neonatal Period: A Systematic Review and Meta-Analysis. Int J Nurs Stud. 2019;92:97–108.

[25] Teune MJ, van Wassenaer AG, Malin GL, Asztalos E, Alfirevic Z, Mol BWJ, Opmeer BC. Long-Term Child Follow-up after Large Obstetric Randomised Controlled Trials for the Evaluation of Perinatal Interventions: A Systematic Review of the Literature. BJOG. 2013;120:15–22.

[26] Weeks AD, Fawcus S. Management of the Third Stage of Labour: (For the Optimal Intrapartum Care Series Edited by Mercedes Bonet, Femi Oladapo and Metin Gülmezoglu). Best Pract Res Clin Obstet Gynaecol. 2020; 67:65–79.

[27] Mattei D, Pietrobelli A. Micronutrients and Brain Development. Curr Nutr Rep. 2019;8:99–107.

[28] Ceriani Cernadas JM. Timing of umbilical cord clamping of term infants. Arch Argent Pediatr. 2017;115:188–194.

[29] Fuwa K, Tabata N, Ogawa R, Nagano N, Yamaji N, Ota E, Namba F. Umbilical Cord Milking versus Delayed Cord Clamping in Term Infants: A Systematic Review and Meta-Analysis. J Perinatol. 2020;.

[30] Marks KP, Madsen Sjö N, Wilson P. Comparative Use of the Ages and Stages Questionnaires in the USA and Scandinavia: A Systematic Review. Dev Med Child Neurol. 2019;61:419–430.
